# Paclitaxel-Induced Collagenous Colitis: A Case Report in Male Breast Cancer

**DOI:** 10.3390/reports8040244

**Published:** 2025-11-24

**Authors:** Shuhei Suzuki, Hidekazu Horiuchi, Takanobu Kabasawa, Takashi Oizumi, Yuka Kobayashi

**Affiliations:** 1 Department of Internal Medicine, Yamagata Prefectural Shinjo Hospital, Shinjo 996-8585, Japan; 2 Department of Pathology, Yamagata University School of Medicine, Yamagata 990-9585, Japan; 3 Department of Pharmacology, Yamagata Prefectural Shinjo Hospital, Shinjo 996-8585, Japan

**Keywords:** paclitaxel, collagenous colitis, drug-induced colitis, chemotherapy toxicity

## Abstract

**Background and Clinical Significance**: Collagenous colitis is an uncommon form of microscopic colitis characterized by chronic watery diarrhea and thickening of the subepithelial collagen layer. While various medications have been implicated in its pathogenesis, paclitaxel-associated collagenous colitis remains exceptionally rare in the literature. Recognition of this adverse event is crucial for appropriate management, particularly in patients receiving dose-modified chemotherapy regimens. This case highlights the importance of considering drug-induced collagenous colitis in cancer patients presenting with severe diarrhea during chemotherapy. **Case Presentation**: We report a 71-year-old Japanese male with metastatic breast cancer who developed acute-onset collagenous colitis during paclitaxel treatment. His primary tumor was invasive ductal carcinoma with hormone receptor-positive, HER2-negative disease (ER+, PgR+, HER2-, Ki-67 46%) and progressive metastatic disease. Given pre-existing renal dysfunction, paclitaxel was initiated at 60% dose reduction. Sixteen days after treatment initiation, the patient experienced abrupt onset of profuse watery diarrhea with approximately 10 bowel movements daily, necessitating hospital admission. Colonoscopic evaluation demonstrated increased vascular permeability and superficial mucosal erosions. Histopathological analysis revealed diagnostic features of collagenous colitis with a markedly thickened subepithelial collagen band measuring 23 μm. Following immediate cessation of paclitaxel, the patient experienced complete resolution of diarrheal symptoms without subsequent relapse. **Conclusions**: This case represents a rare manifestation of paclitaxel-induced collagenous colitis. Clinicians should maintain heightened awareness of this potential complication in patients receiving taxane-based chemotherapy who develop significant diarrhea. Prompt recognition and immediate drug discontinuation are essential for favorable outcomes and symptom resolution.

## 1. Introduction and Clinical Significance

Collagenous colitis is a form of microscopic colitis characterized by chronic watery diarrhea, normal or near-normal colonoscopic appearance, and distinctive histological features including a thickened subepithelial collagen band of 10 μm or greater [1,2]. The condition predominantly affects middle-aged and elderly individuals, with a strong association with certain medications including nonsteroidal anti-inflammatory drugs, proton pump inhibitors, and selective serotonin reuptake inhibitors [3,4].

Paclitaxel is a microtubule-stabilizing agent commonly used in cancer chemotherapy. Taxane-induced colitis has been reported in the literature, with paclitaxel-associated gastrointestinal toxicity occurring in approximately 0.2% of patients receiving taxane-based chemotherapy [5]. The mechanism of taxane-induced colitis is thought to involve direct mucosal toxicity and potential effects on rapidly dividing intestinal epithelial cells [6].

However, specific reports of paclitaxel-induced collagenous colitis are extremely rare in the medical literature. Most reported cases of taxane-induced colitis present as ischemic colitis rather than microscopic colitis [7,8]. This case report describes a rare instance of paclitaxel-induced collagenous colitis, highlighting the importance of recognizing this uncommon but potentially serious adverse event.

## 2. Case Presentation

A 71-year-old Japanese male was referred to our oncology department for treatment of metastatic breast cancer. The patient had no history of tobacco or alcohol use. His family history included gastric cancer in paternal relatives. The patient had multiple comorbidities including hypertension with secondary nephropathy requiring hemodialysis since age 51, previous spinal surgeries, and coronary artery disease.

His medication regimen had remained unchanged for over 7 years and included loxoprofen, pitavastatin, vonoprazan, aspirin, carvedilol, limaprost, precipitated calcium carbonate, lormetazepam, and methyldopa. The patient had no history of previous colonoscopy or colorectal cancer screening despite being over the recommended age for screening.

**Figure 1:**
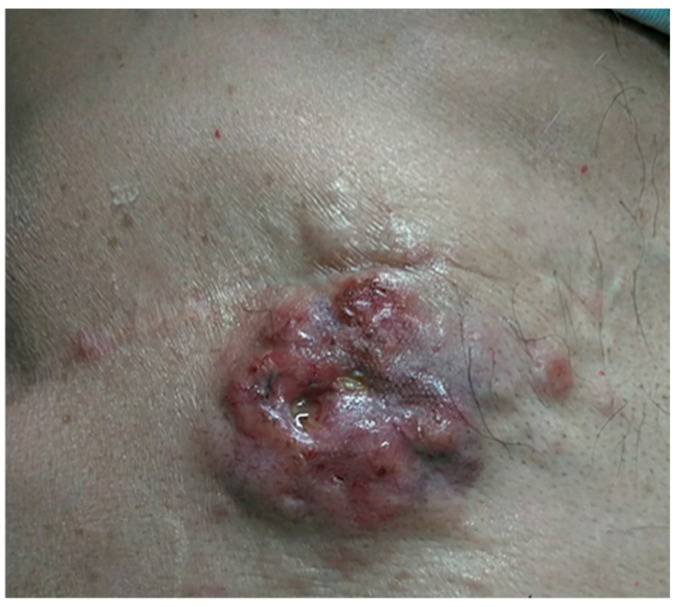
Local findings at initial consultation. Clinical presentation of recurrent breast cancer.

The patient was initially diagnosed with invasive ductal carcinoma of right breast. The tumor was estrogen receptor-positive, progesterone receptor-positive, HER2-negative, and had a Ki-67 proliferation index of 46%. After mastectomy and lymph node dissection, he developed metastatic disease with skin and lymph node involvement (Figure 1). He had received multiple lines of endocrine therapy including anastrozole, letrozole, and fulvestrant with palbociclib, achieving periods of stable disease before eventual progression.

**Figure 2:**
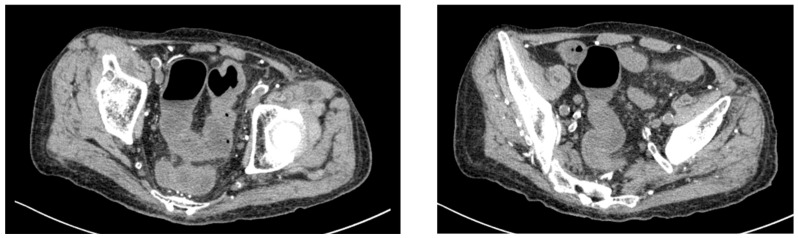
Abdominal computed tomography findings at emergency department presentation. The CT scan demonstrates thickening of the colonic wall.

Given his renal impairment and dialysis dependence, paclitaxel was initiated at a reduced dose of 60% of the standard dose. On day 16 of paclitaxel treatment, the patient experienced severe diarrhea with 10 episodes per day and presented to the emergency department. Computed tomography of the abdomen revealed findings consistent with colitis (Figure 2). Laboratory investigations showed inflammatory changes (Table 1). The patient was admitted for further evaluation and management.

**Figure 3:**
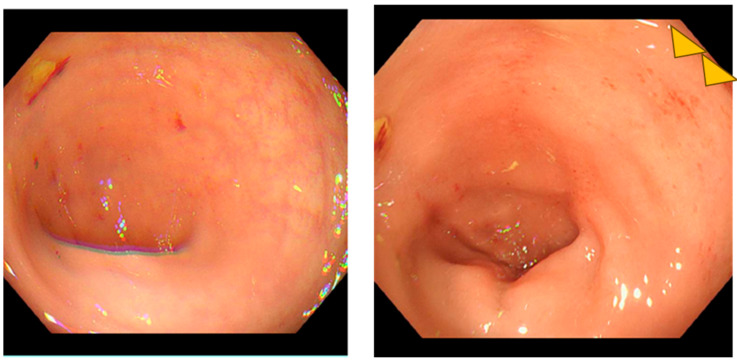
Colonoscopic findings on day 18 of hospitalization. The endoscopic images reveal increased vascular permeability manifested as prominent vascular patterns (yellow triangles).

**Figure 4:**
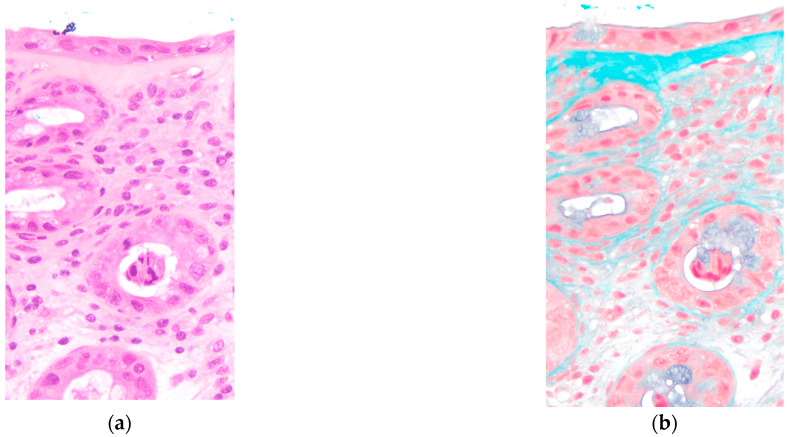
Histopathological findings confirming collagenous colitis (×400): (**a**) Hematoxylin and eosin staining; (**b**) Elastica Masson staining.

Conservative management was initiated with bowel rest, loperamide, and probiotic supplementation. The patient showed partial improvement with diarrhea frequency decreasing to 5 episodes per day by day 18. Lower gastrointestinal endoscopy was performed on day 18, revealing increased vascular permeability and mild erosions in the colon (Figure 3). Histological examination of colonic biopsies demonstrated a markedly thickened subepithelial collagen band measuring 23 μm, significantly exceeding the diagnostic threshold of 10 μm for collagenous colitis. On hematoxylin and eosin staining, eosinophilic homogeneous material was observed in the subepithelial region beneath the surface epithelium. Elastica Masson staining clearly demonstrated the extent of collagen fiber deposition as a continuous blue-stained band. The crypts maintained normal architecture without distortion or atrophy. These histopathological features, combined with the thickened subepithelial collagen band, confirmed the diagnosis of collagenous colitis (Figure 4). Lymphocyte infiltration was scarcely observed in the surface epithelium.

Based on the temporal relationship between symptom onset and drug administration, along with the patient’s clinical course, paclitaxel was identified as the most likely causative agent. Paclitaxel was immediately discontinued upon diagnosis. The patient’s symptoms resolved rapidly, with complete resolution of diarrhea by day 4 of hospitalization. He was discharged on hospital day 14 after coordination with the hemodialysis schedule.

Comprehensive infectious workup including cytomegalovirus and *Clostridioides difficile* toxin assays were negative. Stool cultures revealed no significant pathogenic organisms. Paclitaxel rechallenge was not attempted given the severity of this adverse event. Over the subsequent 4 months of follow-up, the patient experienced no recurrence of diarrheal symptoms.

Cancer genomic profiling was performed and revealed a somatic *BRCA2* deletion, with germline testing negative for *BRCA1/2* mutations. Based on this finding, the patient was initiated on olaparib therapy, which has demonstrated sustained efficacy with maintained response at the time of this report.

## 3. Discussion

This case represents a rare instance of paclitaxel-induced collagenous colitis in a male breast cancer patient. The diagnosis was established through the characteristic clinical presentation of severe watery diarrhea, typical histological findings including a thickened subepithelial collagen band measuring 23 μm, and the temporal relationship with paclitaxel administration.

Collagenous colitis is characterized by a thickened subepithelial collagen band of 10 μm or greater [1,2]. Our patient’s collagen band thickness of 23 μm clearly exceeded this diagnostic criterion. The normal subepithelial collagen layer is approximately 3 μm in thickness [9].

Drug-induced collagenous colitis has been strongly associated with several medication classes, particularly non-steroidal anti-inflammatory drugs (NSAIDs) and proton pump inhibitors [3,4]. A case–control study found that long-term NSAID use (greater than 6 months) was significantly more common in collagenous colitis patients than controls [10].

Taxane-induced colitis has been reported in the literature. A comprehensive retrospective study of 45,527 patients receiving taxane therapy identified 76 cases (0.2%) of taxane-induced colitis [5]. The median time from treatment initiation to symptom onset was 31 days [5], which is consistent with our patient’s presentation on day 16. However, most reported cases involved ischemic colitis with endoscopic evidence of mucosal ulceration, rather than microscopic colitis [7,8,11]. Chemotherapy-induced intestinal microbiota alterations contribute to drug-induced diarrhea. Topoisomerase I inhibitors, particularly irinotecan, and taxanes induce diarrhea through direct intestinal toxicity and microbiota disruption. However, the marked subepithelial collagen deposition in our patient indicates a specific drug-induced collagen synthesis response rather than solely microbiota-mediated effects. The combination of microbiota alterations and direct drug effects on colonic tissue likely contributed to the development of collagenous colitis.

In our patient, vonoprazan, a proton pump inhibitor, was considered as a potential causative agent. Proton pump inhibitors have been associated with microscopic colitis in multiple studies [12]. However, the temporal relationship strongly supported paclitaxel as the causative agent. Symptom onset occurred 16 days after paclitaxel initiation, while vonoprazan had been used unchanged for over 7 years without prior symptoms. Vonoprazan and other medications were promptly restarted after resolution of diarrhea, with no recurrence of colitis observed.

The management of drug-induced collagenous colitis primarily involves discontinuation of the offending agent. Our patient’s rapid clinical improvement following paclitaxel discontinuation, without the need for specific anti-inflammatory therapy, demonstrates the importance of early drug recognition and discontinuation. Several limitations should be acknowledged in this case report. First, it is challenging to assess whether the patient’s concurrent dialysis status may have influenced the development or clinical course of the colitis itself or specifically contributed to the pathogenesis of paclitaxel-induced colitis. Second, the exclusion of culture-negative toxin-mediated colitis remains difficult, as conventional bacterial culture methods may fail to detect certain toxin-producing pathogens that could present with similar clinical manifestations. The precise mechanism underlying paclitaxel-induced collagen deposition remains unclear.

An important differential diagnosis in this patient is chronic uremic colitis associated with his long-term hemodialysis status. Chronic uremic colitis typically presents with colonoscopic findings of mucosal ulcerations and hemorrhagic changes, distinct from the microscopic findings observed in collagenous colitis. In our patient, colonoscopy revealed only increased vascular permeability and superficial erosions without typical uremic colitis findings. Furthermore, the acute onset of symptoms 16 days after paclitaxel initiation, rather than a chronic or progressive course, strongly suggested drug-induced etiology rather than uremia-related changes. The patient had been on stable hemodialysis for 20 years without prior gastrointestinal symptoms, which further supports paclitaxel as the causative agent rather than his underlying renal condition.

This case highlights a rare but serious adverse event that clinicians should consider in patients receiving taxane-based chemotherapy who develop severe diarrhea. Early recognition and drug discontinuation would be important for optimal outcomes.

## 4. Conclusions

We report a rare case of paclitaxel-induced collagenous colitis in an elderly male patient with metastatic breast cancer. This adverse event presented with severe watery diarrhea and characteristic histological findings, including a significantly thickened subepithelial collagen band measuring 23 μm. The patient’s symptoms resolved rapidly following paclitaxel discontinuation, supporting a causal relationship.

This case emphasizes the importance of maintaining awareness of drug-induced collagenous colitis in patients receiving chemotherapy, particularly in elderly patients with multiple comorbidities. Clinicians should consider collagenous colitis in the differential diagnosis of severe chemotherapy-induced diarrhea and recognize that early drug discontinuation is essential for optimal outcomes.

## Figures and Tables

**Table 1:** Laboratory data at emergency department visit.

Biochemistry			Immunology		
TP	5.6	g/dL	CRP	10.95	mg/dL
Alb	2.7	g/dL			
AST	22	U/L	Hematology		
ALT	16	U/L	WBC	2140	/uL
T-Bil	0.41	mg/dL	Neutrophils	67.2	%
LDH	241	U/L	Lymphocytes	15	%
ALP	77	U/L	Monocytes	7	%
Cre	5.95	mg/dL	Eosinophils	10.3	%
UN	29.4	mg/dL	Basophils	0.5	%
Na	141	mmol/L	Hb	7.0	g/dL
K	4.2	mmol/L	Plt	15.8	10^4^/uL
Cl	103	mmol/L			

TP, total protein; Alb, albumin; AST, aspartate aminotransferase; ALT, alanine aminotransferase; T-Bil, total bilirubin; LDH, lactate dehydrogenase; ALP, alkaline phosphatase; Cre, creatinine; UN, urea nitrogen; CRP, C-reactive protein; WBC, white blood cell count; Hb, hemoglobin; Plt, platelet count.

## Data Availability

The data presented in this study are available within the article. Additional data are not publicly available due to patient privacy and confidentiality restrictions.

## Abbreviations

The following abbreviations are used in this manuscript:

NSAID	non-steroidal anti-inflammatory drug
